# The guiding strength of sensory templates during preparatory stage of sustained feature-based attention

**DOI:** 10.1080/13506285.2026.2687589

**Published:** 2026-06-15

**Authors:** Xiaoli Zhang, Taosheng Liu

**Affiliations:** Department of Psychology, Michigan State University, East Lansing, MI, USA

**Keywords:** Attentional templates, template-driven capture, sensory templates, working memory, preparatory attention

## Abstract

Visual attention is guided by attentional templates that store task-relevant information, but whether these templates contain sensory details, and how task factors influence their content and guiding strength remains unclear. We investigated these questions by combining sustained feature-based attention with a search task containing an irrelevant distractor that either matched or mismatched the target color in the sustained attention task. The difference in attentional capture by the distractor in the same-color versus different-color condition indexed the strength of the sensory template. We manipulated selection demand by varying the similarity between the two colors in the sustained attention task and presented cues in either sensory or non-sensory formats. With sensory cues, capture magnitude remained equally strong under both low and high selection demand. However, with non-sensory cues, low selection demand attenuated but did not fully eliminate capture. These results demonstrate that attentional templates store sensory content of cued features. The guiding strength of templates is not susceptible to selection demand with sensory cues, but non-sensory cues delayed or reduced the activation and maintenance of sensory templates under low selection demand. This suggests that cue format and selection demand contribute to how sensory information is represented in attentional templates to guide selection.

We are immersed in a world with an enormous amount of visual information. One critical function of the human cognitive system is attention, which selects and prioritizes information relevant to current behavioural goals. In many situations, attentional selection is guided by known target features, such as the color blue when searching for a blue dress in a clothing store (Wolfe, 2021). Prominent theories of attention propose that feature-based selection is mediated by attentional templates, which are internal representations of target features maintained in working memory (Desimone & Duncan, 1995; Duncan & Humphreys, 1989; Treisman & Gelade, 1980; Wolfe, 2021). However, a fundamental question remains regarding how these attentional templates are mentally represented.

Although it may be too rigid to assume that attentional templates are always picture-like representations, many studies have indicated that attentional templates contain sensory information of the target. For example, Vickery et al. (2005) found that search performance scaled with the degree of visual match between cue and target, and proposed that setting up the target template uses visual rather than semantic information. Similarly, picture cues are found to be more effective than word cues in guiding search in real-world scenes (Malcolm & Henderson, 2009), and visual similarity, measured independently of object categories, continues to guide attention even when search is performed for categories (Alexander & Zelinsky, 2011).

However, other studies show that attentional templates do not solely contain the same information as perceptual representations and may not always be sensory while maintained in working memory. Instead, they may contain multiple types of information and could vary due to task demands. For example, de Groot et al. (2016) found a substantial oculomotor bias towards semantically related objects during visual search, indicating the target template contains semantic information. Research on attending to object categories further demonstrates that attentional templates can be viewpoint-independent (Reeder & Peelen, 2013), can be formed on different hierarchical levels of the categories (Maxfield & Zelinsky, 2012), and can have category tuning breadth flexibly adjusted to task demands (Addleman et al., 2024), all suggesting that these attentional templates are somewhat flexible and not purely sensory in nature.

A question that naturally arises from these studies is: what factors influence how attentional templates are represented? More specifically, what factors influence whether the templates contain sensory information, say color features, and how strongly they guide attention? To answer this question, we took inspiration from the research on working memory-driven capture, which showed that information maintained in working memory captures attention even when it is presented as an irrelevant distractor during a secondary search task (Soto et al., 2005). First, this memory-driven capture effect has been widely replicated and used as a behavioural measure to indirectly assess the representation of memorized features (e.g., Baier & Ansorge, 2019; Kerzel, 2019; Olivers et al., 2006; Sun et al., 2015) and even the representation of task-irrelevant information (such as Doyle et al., 2025; Dube & Golomb, 2021). The general logic here is that to the extent that a distractor with memorized feature captures attention and impairs search performance, it indicates that the memorized feature is actively held in mind. We adopted this inferential logic to examine whether there are sensory (color) components in attentional templates (henceforth referred to as “*sensory templates*” in this paper), given that templates are believed to be actively maintained in working memory representations (Desimone & Duncan, 1995; Wolfe, 2021). Second, using this paradigm, Olivers et al. (2006) found the memory-driven capture effect when the task required encoding of visual details (e.g., a specific shade of red compared with other reds), but not when the task required only coarse, verbal encoding (e.g., red compared with other categorically different colors). This finding reveals memory demand – the required precision of memory retrieval – is one critical factor influencing the degree of sensory detail encoded in working memory representations. Given the tight link between working memory and attentional templates (Desimone & Duncan, 1995; Wolfe, 2021), it is possible that a similar manipulation of task demand would have an analogous effect on attentional templates. One such manipulation in attention research is selection demand, often operationalized by varying target-distractor similarity in visual search tasks. Selection demand has long been shown to influence search behaviour, with higher target-distractor similarity resulting in worse performance (e.g., Calder-Travis & Ma, 2020; Duncan & Humphreys, 1989). High selection demand is also considered to entail more precise templates (Geng et al., 2017; Kerzel, 2019). However, less is known about the sensory nature and the guiding strength of templates under different selection demands, as has been shown in the domains of working memory. Would selection demand change how sensory the templates are, and the strength of guidance provided by the sensory templates?

In addition to selection demand, another important factor may have been overlooked in previous studies on attentional templates. Experimental paradigms of attention often use cues that contain the exact sensory information as the target (e.g., a red circle cue for searching red targets). But in real life, when we need to search for something (e.g., a friend asks for help finding their mug), we almost never have the exact sensory cue available (e.g., an exact image of that mug); instead, we have a verbal description such as “red mug.” How we form attentional templates after receiving non-sensory cues is an ecologically important but under-investigated question. Some studies have investigated whether non-sensory cues produce sensory templates, but a consistent picture has yet to emerge. For example, Sun et al. (2015) showed that semantic word cues automatically induced corresponding color capture even when color was task irrelevant (e.g., “rose” induced capture by red and “sea” by blue). On the other hand, Baier and Ansorge (2019) found that color-word cues only induced word-based capture but not color-based capture. Thus, it remains unclear whether sensory templates are typically formed after receiving non-sensory or semantic input, and if so, whether selection demand plays an adaptive role in such transformation.

Therefore, our current study aims to investigate whether selection demand and cue format influence how much sensory information is contained in attentional templates and how much guiding strength sensory templates provide. We chose to test color-based templates because color is perhaps the most widely studied feature in visual cognition and is known to be a reliable and powerful feature for attentional guidance (Anderson et al., 2010; Huang, 2015; Hulleman, 2020; Teng & Kravitz, 2019). While most of the research on attentional templates has used visual search tasks, visual search is only one of the tasks in which attention plays a role. Visual search is also a complex task that often involves multiple shifts of attention and is influenced by decision strategies (e.g., quitting threshold; Wolfe, 2012) and may involve different mechanisms during search guidance and target-match decision processes (Kerzel, 2019; Yu et al., 2022). In order to isolate and investigate the properties of attentional templates maintained during preparation, we employed a sustained feature-based attention task as the participants’ main attention task. We manipulated selection demand by varying target and non-target color similarity in the sustained attention task while carefully controlling for task difficulty to maintain a consistent level of task engagement. We also manipulated cue format with sensory versus non-sensory (word) cue presentations to investigate how cue format influences template representation.

We examined template representations using a design modified from the working memory-driven capture paradigm (Olivers et al., 2006). This allows us to assess both the *existence* and *strength* of sensory template representations during the preparatory stage at the behavioural level by measuring the amount of capture by a distractor that matches the content of the template. Specifically, there are three distractor conditions for this search task: no distractor (“baseline”), distractor with a color very different from the attended color (“different color”), and distractor with the same color as the attended color (“same color”). By measuring the magnitude of *salience-driven capture* (i.e., the response time difference between different-color and baseline conditions), we can evaluate how much guidance the overall color dimension provides; by measuring the existence and magnitude of *template-driven capture* (i.e., the response time difference between same-color and different-color conditions), we can evaluate whether the template contains a sensory component and how much top-down guidance the sensory template of the target feature provides. Considering that salience-driven capture is primarily a bottom-up process, we do not expect selection demand or cue format to change its magnitude with overall task engagement being constant. Critically, following analogous results from working memory (Olivers et al., 2006), we may expect selection demand to modulate the magnitude of template-driven capture. Sensory cue format may further enhance the capture due to the exact sensory information. On the other hand, if we do not observe such modulations of demand or cue format, it would imply a relatively constant strength of attentional guidance, indicating persistently active sensory templates during preparatory attention. Either outcome would be informative regarding the nature of attentional templates for visual features.

## Experiment 1

In the first experiment, we manipulated selection demand in a within-subject design by varying the similarity of the target and distractor colors in a feature-based attention task. On half of the trials, participants performed this feature-based attention task, and on the other half of the trials, they performed a shape search task where the target color of the feature-based attention task could appear on a distractor. This design with two tasks randomly alternating on each trial makes it impossible for participants to predict the next trial and develop strategies such as task switching, thus allowing us to assess the template representation when they need to actively maintain the cued color on every trial.

### Methods

#### Transparency and openness

We report our sample size decision, all data exclusions, all manipulations, and all measures in the study. All experiment code, data, and analysis code are available online (https://osf.io/6zqmy).

#### Participants

We predefined and preregistered our sample size to be 30 (https://osf.io/skwz4). This was based on a power analysis of a pilot dataset which showed a content-driven capture effect (data not included in this report). Participants were Michigan State University undergraduate students, who gave informed consent and were compensated with course credits. All participants reported normal or corrected-to-normal vision and normal color vision. The study protocols for all of our experiments were approved by the Institutional Review Board at Michigan State University.

Thirty-one participants completed Experiment 1. Data from one participant was excluded due to very long search task response times (mean RT; greater than 3 standard deviations from the sample group mean). The remaining 30 participants were included in the analyses (19 females, 9 males, 2 other genders, age mean = 19.08, SD = 1.12).

#### Apparatus and stimulus

Stimuli were generated using MATLAB (The Mathworks Inc., 2015) and MGL (Gardner et al., 2018), and presented on a 21-in. CRT monitor (1024 × 768 pixels; 75 Hz refresh rate). Participants were seated in a dark room at a viewing distance of 73 cm.

All colors were selected from an isoluminant color wheel in the CIE L*a*b space (L = 73, a = 7, b = 17, r = 91), obtained by calibrating the display monitor using an I1 Pro spectrophotometer (X-Rite Inc. Grand Rapids, MI, USA). In Experiment 1, attended colors were selected from 4 categories: red, yellow, green, and blue, with three exemplars in each category. These colors were selected by the experimenter to balance the distinctiveness and variability across exemplars in each color category. The converted RGB values for the 12 colors are reported in Supplemental Table S1.

#### Design and procedure

**Figure 1.**
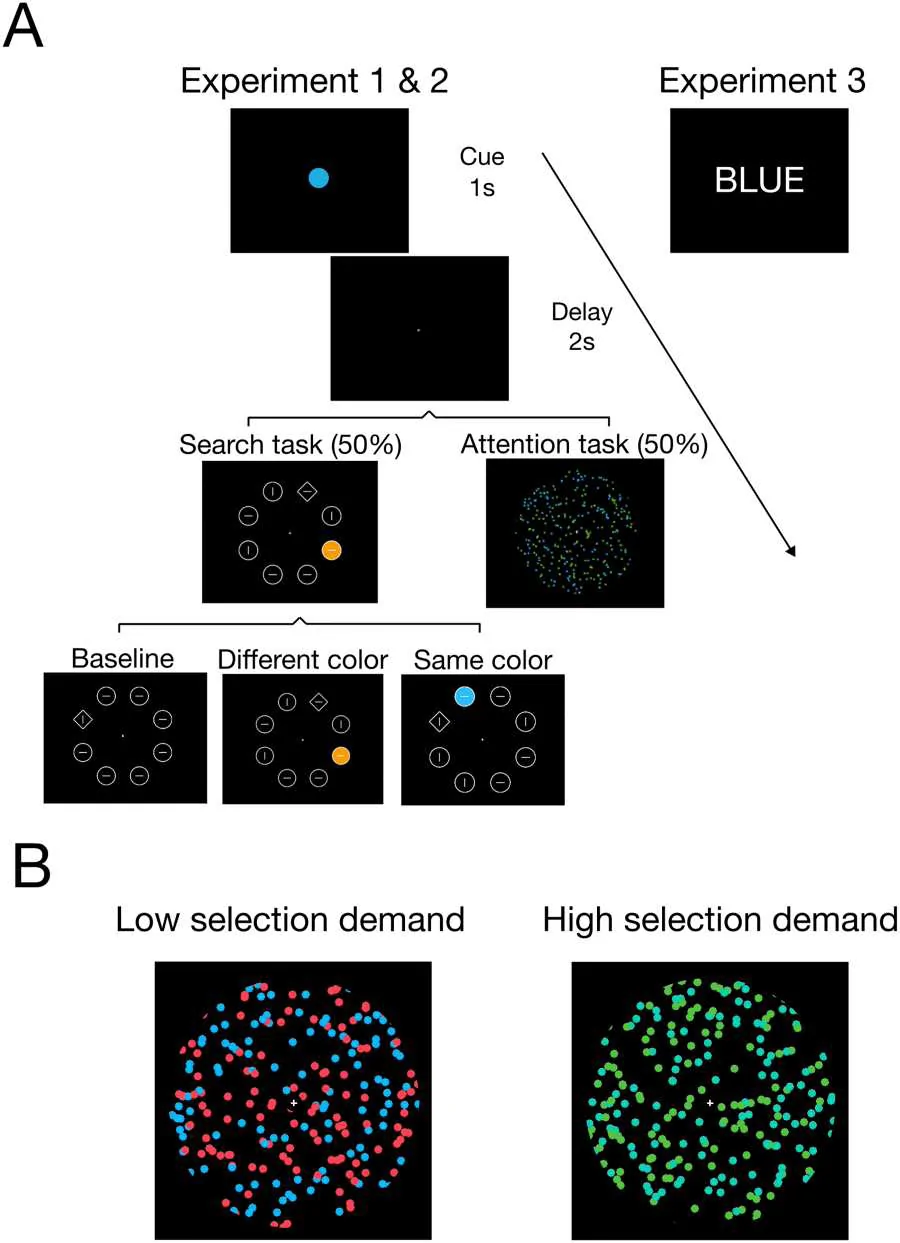
(Color online) Illustration of experimental paradigms. (A) Example trial structures for all experiments. Images do not reflect the actual scale of the stimuli. (B) Example stimulus display in the Attention task for low (120° difference) and high (40° difference) selection demand (dot size is enlarged for illustration purpose).

In Experiment 1, each trial started with a color circle (diameter: 2.4°) at the centre of the screen for 1s. The color was randomly chosen from the 12 possible target colors and participants were instructed to remember this color cue in preparation for the subsequent task. After a 2s delay, one of two tasks was chosen randomly with equal probability (Figure 1), as described below.

In the sustained feature attention task (i.e., the Attention task), a dot field in the cued color and one distractor color would be presented for 2s. The dot field was presented at the screen centre (aperture diameter: 12°; dot size: 0.1°; dot density: 2.5 dots/deg^2^). Half of the dots were randomly chosen to be in the cued color, and the other half were in the distractor color; they also moved slowly with random directions (Brownian motion) at a speed of 1°/s. Participants were asked to pay attention to the cued-color dots and report whether there was a dimming event (duration: 0.2s) among them. Three types of events could occur with equal likelihood: dimming among cued-color dots, dimming among distractor-color dots, and no dimming. Dimming events would only occur in a 0.5– 1.5s window during the dot field presentation and dimming only occurred for a randomly chosen 50% of dots in the designated color. This was done to prevent temporal and spatial predictability of the dimming events, so that participants needed to use sustained feature attention to perform the task. After the dot field display was removed from the screen, participants were instructed to press the “x” key on the keyboard to report dimming among cued-color dots and press the “z” key for no dimming among cued-color dots. The maximum response time was 3s.

On the other half of the trials, participants performed a visual search task (i.e., the Search task). After the delay, an array of 8 shape outlines (white color; line thickness: 0.1°) would be presented along an imaginary circle (eccentricity: 5.5°). Among the search items, 7 of them would be circles (diameter: 2.4°) and 1 diamond (diagonal: 2.4°). Each shape had a randomly selected horizontal or vertical line at the centre (white color; length: 1°; line width: 0.1°). Participants were instructed to search for the diamond shape and report the orientation of the line in it, by pressing the left arrow key for horizontal and the down arrow key for vertical. There were three critical conditions in the Search task. For the “baseline” condition, none of the shape outlines were filled with color; for the “same color” condition, one of the circles was filled with the cued color; for the “different color” condition, one of the circles was filled with a color that was 120° away from the cued color on the color wheel. These three conditions were equally likely (^1^/_3_ each of the Search trials) and randomly interleaved. The locations of the diamond target and that of the color distractor were also randomized. Participants were explicitly told that the color cued at the beginning of trial was not relevant for the Search task. After the onset of the search display, participants had a maximum of 3s to make a key press response while the search display remained on the screen.

For either the Attention or the Search task, if participants failed to make a response within the time limit or made an incorrect response, they would receive error feedback via a beep sound with “Time is up!” or “Incorrect!” text on the screen. Trials for the Attention and the Search task were randomly interleaved within each block, so that during the cue and the delay period participants could not predict which task would follow.

We manipulated the selection demand – high and low – of the Attention task by implementing staircase procedures on different parameters of the stimuli in separate blocks. In the low-demand condition, the uncued color was kept at 120° away from the cued color on the color wheel (clockwise or counterclockwise, randomly chosen each trial), making the two colors highly distinct (Figure 1(B) left). To control for task difficulty, we adjusted the magnitude of the dimming – the proportion of change in RGB values – with a staircase, setting the intended accuracy level at 75% – 80% correct. In the high-demand condition, we kept the magnitude of dimming at a high and easy level of 70% and adjusted the angular distance on the color wheel between the cued and the uncued colors (Figure 1B right), again setting the intended accuracy level at 75% – 80%. The staircases were controlled by the Best PEST algorithm as implemented in the Palamedes Toolbox (Prins & Kingdom, 2018), separately for each of the four color categories to account for potential categorical differences. The staircase procedures thus created high and low-demand conditions by varying the similarity of the cued and uncued color, while keeping the overall task difficulty to be comparable. These staircases were continuously updated throughout the experiment to maintain a steady level of task difficulty and keep participants engaged.

Participants performed 6 blocks of the low-demand condition in the first half of the experiment and 6 blocks of the high-demand condition in the second half, or in the opposite order, counterbalanced across participants. In each half, they first practiced the tasks for at least 15 trials until they understood and achieved reasonable performance (i.e., five correct trials consecutively). Each block contained 27 trials of the Search task and 27 trials of the Attention task, yielding 162 trials for each task in each demand condition.

#### Analysis

Data were processed using a custom script with MATLAB, and statistical tests were performed using JASP (JASP Team, 2024). For simplicity, we reported ANOVAs with null hypothesis significance testing (NHST) and reported post hoc t-tests with both NHST and Bayesian statistics (*BF*_*10*_ or *BF*_*01*_).

Participants with very low accuracy in the Attention task (below 60%) or very long RT in the Search task (greater than 3 standard deviations from the sample group mean) were excluded as outliers. This resulted in 1 participant being excluded and N = 30 included in the following analyses. Further, we excluded Search trials which had RT shorter than 200 ms or outside of 3 standard deviations from the mean RT for each condition within each participant, resulting in the exclusion of 1.8% of the Search trials in total.

For data visualization purposes, within-subject error bars are calculated (Cousineau, 2005; Morey, 2008) and plotted in all figures.

#### Results and discussion

With our staircase procedures, the stabilized hue difference between cued and uncued colors in the high demand condition was on average below 30° on the color wheel, much smaller than 120° used in low demand, and the resulting attention task accuracy was about 77–80% and comparable between high and low demand conditions (detailed results shown in Supplemental Figure S1). This confirms successful manipulation of selection demands while maintaining overall task engagement across both conditions.

**Figure 2.**
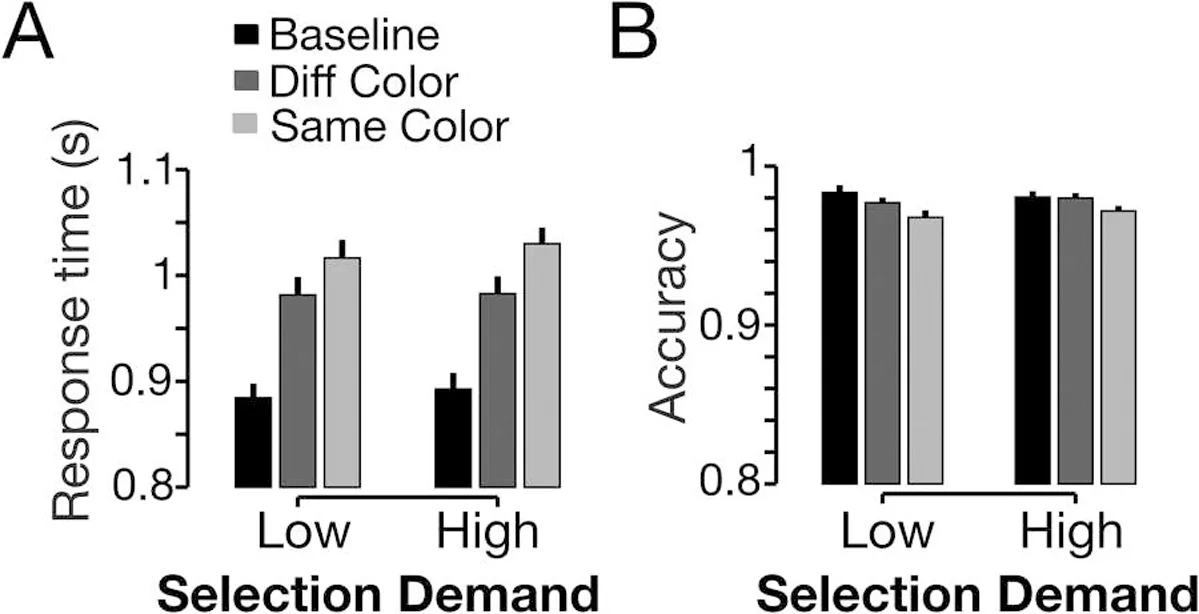
Search task results of Experiment 1. (A): Search task response time for baseline, different-color, same-color distractor conditions and for low and high demand conditions. (B) Search task accuracy for each distractor condition and each demand condition. Within-subject error bars are plotted.

Because selection demand may have differential effects on salience-driven and template-driven capture, we assessed these two sources of capture effects in separate analyses. We first investigated the capture effect driven by the salient distractor and the impact of selection demand (Figure 2). We submitted the Search task RT data from correct trials into a 2 (high vs. low selection demand) by 2 (different-color vs. no distractor baseline) repeated-measures ANOVA. There was a significant main effect of distractor status, *F*(1, 29) = 131.624, *p* <.001, $\eta_{p}^{2}=.819$, with the different-color condition having longer RT compared with the baseline. No main effect of selection demand nor interaction was found (*F*(1, 29) = 0.031, *p* = .861, $\eta_{p}^{2}=.001$; *F*(1, 29) = 0.381, *p* =.542, $\eta_{p}^{2}=.013$). To further assess the lack of main effect and interaction from selection demand, we examined the magnitude of salience-driven capture by calculating the RT difference between the different-color and baseline conditions, separately for low and high demand. Paired samples t-test on salience-driven capture magnitude showed substantial evidence for no difference between high and low demand (*t*(29) = 0.617, *p* = .542, *Cohen’s d* = 0.113, *BF*_*01*_ = 4.315).

Our primary interest is using the Search task performance to assess the template representation during the preparatory stage. For this purpose, we investigate the capture effect driven by attentional templates, and submitted the data to a 2 (high vs. low selection demand) by 2 (different-color vs. same-color condition) repeated-measures ANOVA. There was a significant main effect of distractor color, *F*(1, 29) = 25.479, *p* <.001, $\eta_{p}^{2}=.468$, showing the same-color condition having longer RT compared with the different color condition. We did not find a significant main effect of demand nor interaction (*F*(1, 29) = 0.067, *p* = .797, $\eta_{p}^{2}=.002$; *F*(1, 29) = 1.177, *p* =.287, $\eta_{p}^{2}=.039$). Paired samples t-test on template-driven capture magnitude (i.e., same-color RT minus different-color condition) was performed between selection demand conditions and revealed substantial evidence for no difference (*t*(29) = 1.085, *p* = .287, *Cohen’s d* = 0.198, *BF*_*01*_ = 3.010).

We also analyzed the accuracy of the Search task and ruled out the contribution of potential speed-accuracy tradeoff to the observed RT effects. The group-averaged accuracy of the Search task was close to the ceiling (~97% and above). We submitted the accuracy data into the same ANOVAs as those performed with RT data. The first ANOVA (high vs. low selection demand by different-color vs. no distractor baseline) did not show any main effects or interaction, *Fs* ≤ 0.728, *p* ≥.401 $\eta_{p}^{2}s\leq .024$. In the second ANOVA (high vs. low selection demand by different-color vs. same-color condition), we found that the same-color condition had lower accuracy compared to the different-color condition, which aligned with the RT result showing worse performance for the same-color than different-color conditions (*F*(1, 29) = 7.938, *p* = .009, $\eta_{p}^{2}=.215$). There was no main effect of selection demand or interaction, *Fs* ≤ 0.752, *p* ≥.393, $\eta_{p}^{2}s\leq .025$.

Taken together, there was a salience-driven capture effect where a singleton distractor of a different color increased Search RT compared to the no distractor baseline. When the distractor color matched the attentional template, we found Search performance further impaired by template-driven capture – reflected in both longer RT and lower accuracy – compared to a distractor of a different color. We did not find selection demand modulating the strength of the salience-driven capture effect, nor the strength of the template-driven capture effect.

In Experiment 1, each participant performed the low and high demand conditions in a specific order (e.g., low demand in the first half and high demand in the second half, or vice versa). Although the order was counterbalanced across participants and all results above were averaged across task orders, it is still possible that the behavioural patterns or strategies for the second half were influenced by the first half to some extent. Experiment 1 was not designed to test the order effect, and we do not have enough power to compare between subsets of participants and conditions. Therefore, we adopted a between-subject design in the following experiments to rule out this potential confound.

## Experiment 2

In Experiment 2, we investigated if selection demand modulates template-driven capture using a similar paradigm as Experiment 1, but with demand levels manipulated in a between-subject design, in order to eliminate the potential confound of condition order. We collected data from two groups of participants; each group performed the Attention task in either the high selection demand condition or the low selection demand condition.

### Methods

#### Participants

A total of 62 undergraduate students from Michigan State University participated for course credits. Two participants were excluded due to either very low Attention task accuracy (below 60%) or very long Search task RT (greater than 3 standard deviations of the corresponding group mean). The remaining participants’ data, N = 30 in each group, were included in the analyses (43 females, 17 males, age mean = 19.08, SD = 1.63).

#### Apparatus and stimulus

The equipment was identical to that of Experiment 1. We made a small change for the color selection. Using the same isoluminant color wheel as Experiment 1, we pre-defined 6 target colors: magenta, red, orange, yellow, green, blue. Each color category only had one exemplar, and the converted RGB values are reported in Supplemental Table S1. We made this change to target colors to facilitate the word cue manipulation in Experiment 3.

#### Design and procedure

The design and procedure were identical to those of Experiment 1, except that each participant only performed in one selection demand condition (low or high). Each participant completed 6 blocks, with each block containing 27 trials of the Search task and 27 trials of the Attention task, which provided the same number of trials in each demand condition as in Experiment 1. We performed similar analyses to Experiment 1, with the same trial exclusion criteria. A total of 1.6% of the search trials were excluded from analyses due to RT shorter than 200 ms or outside of 3 standard deviations from the mean RT for each condition within each participant.

#### Results and discussion

The overall performance of the Attention task was again comparable between low and high demand conditions (77– 78%). The staircased hue differences between cued and uncued colors in the Attention task in the high demand condition were no more than 40°, much smaller than 120° used in low demand, confirming again that our manipulation of selection demand was successful.

**Figure 3.**
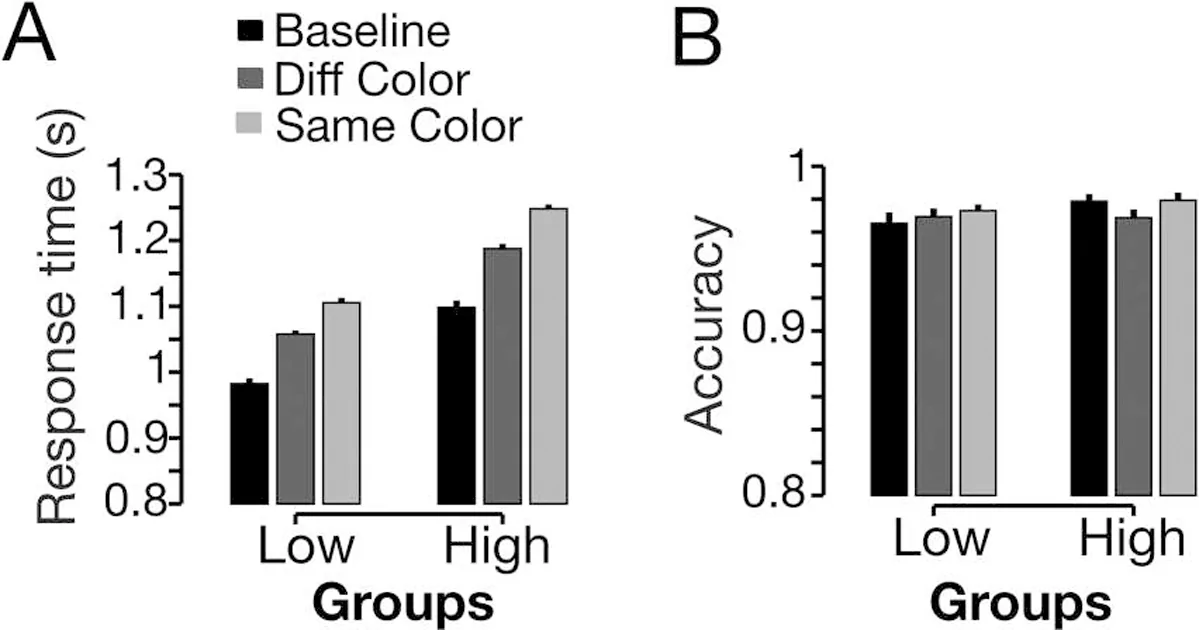
Search task performance of Experiment 2, including response time (A) and accuracy (B) for baseline, different-color, same-color distractor conditions and for Low and High selection demand conditions. Within-subject error bars are plotted.

We first evaluated the salience-driven capture effect by comparing search performance in the baseline and different-color distractor condition and found reliable RT differences in both low and high demand groups (Figure 3). We submitted the RT on correct trials to a 2 (baseline vs. different-color, within-subject) by 2 (low vs. high, between-subject) mixed-factor ANOVA and found a significant main effect where the different-color condition had longer RT than baseline, *F*(1, 58) = 9.560, *p* <.001, $\eta_{p}^{2}=.612$. There was also a significant main effect of selection demand (*F*(1, 58) = 4.886, *p* = .031, $\eta_{p}^{2}=.078$). Given that the baseline condition alone (which should not be influenced by selection demand due to a lack of any color distractor) exhibited a significant group difference in the same direction (*t*(58) = 2.023, *p* = .048, *Cohen’s d* = 0.552; *BF*_*10*_ = 1.425), this main effect of groups likely reflected a sample difference in overall RT instead of a genuine effect of selection demand per se. There was no significant interaction (*F*(1, 58) = 0.640, *p* = .427, $\eta_{p}^{2}=.011$). The post hoc independent samples t-test provided anecdotal evidence for no difference in the magnitude of salience-driven capture between low and high demand groups (*t*(58) = 0.800, *p* = .427, *Cohen’s d* = 0.207, *BF*_*01*_ = 2.912). We performed similar AVONAs on accuracy, and there were no significant main effects nor interactions, *Fs* ≤ 1.508, *ps* ≥.224, $\eta_{p}^{2}s\leq 0.025$.

To investigate the template-driven capture effect in the Search task, we focused on the comparisons between same-color and different-color distractor conditions and again found RT differences in both low and high demand groups. We submitted the RT on correct trials to a 2 (same- vs. different-color, within-subject) by 2 (low vs. high, between-subject) mixed-factor ANOVA. We found a significant main effect of distractor color, *F*(1, 58) = 90.515, *p* <.001, $\eta_{p}^{2}=.609$, showing longer RT in the same-color condition compared to the different-color condition. There was also a significant main effect of groups where RT was longer in the high demand group than in the low demand group, *F*(1, 58) = 5.959, *p* = .018, $\eta_{p}^{2}=.093$. The interaction between distractor color and selection demand was not significant (*F*(1, 58) = 1.278, *p* = .263, $\eta_{p}^{2}=.022$), and there was anecdotal evidence for no difference in template-driven capture magnitude between the low and high groups (independent samples t-test *t*(58) = 1.131, *p* = .263, *Cohen’s d* = 0.292, *BF*_*01*_ = 2.231). Similar ANOVAs on accuracy did not show significant main effects nor interactions, *Fs* ≤ 2.682, *ps* ≥.107, $\eta_{p}^{2}s\leq 0.044$.

Taken together, we found both template-driven capture and salience-driven capture effects, reflected by RT differences. The result patterns of Experiment 2 replicate those of Experiment 1, where high and low selection demand showed comparable magnitudes of capture effects, indicating a similar strength of sensory templates insusceptible to selection demand levels.

The lack of selection demand effect on template-driven capture magnitude is in apparent contradiction to what Olivers et al. (2006) found for the working memory-based attentional capture effect. In their Experiment 2, participants were explicitly instructed to remember “the global color / more verbally,” where memory probes of the recognition task were from different color categories, and to remember “the precise color / more visually,” where the probes were from the same category. Their results showed that “more visual” encoding led to memory-driven capture whereas “more verbal” did not. It is possible that the explicit instruction played a critical role in inducing working memory content difference and led to different capture magnitude. To test this possibility, we ran an additional experiment of the low selection demand condition only, where participants were explicitly told to hold the attentional template using corresponding color words (results are shown in Supplemental Figure S2). Under this verbal coding instruction, there was still a significant template-driven capture effect, and the magnitude of this capture (i.e., same color minus different color condition) was similar to both high and low demand in Experiments 1 and 2. This finding indicates that “verbal coding” instruction itself did not discourage sensory templates. Such discrepancy between our results and the working memory literature is further discussed in General Discussion.

## Experiment 3

Experiments 1 and 2 showed that even in a low selection demand condition, there was still a strong sensory template maintained such that it produced reliable template-driven capture, and the strength of the sensory template was not susceptible to selection demand. Note that in both experiments, cues for the Attention task were always given in the sensory format. Does the sensory information contained in the cue itself drive such consistent sensory templates? If so, would non-sensory cue formats, like color words, encourage weaker sensory or even non-sensory templates? Experiment 3 aims to investigate whether selection demand modulates attentional templates transformed from non-sensory cue inputs.

### Methods

#### Participants

A total of 61 undergraduate students from Michigan State University participated in Experiment 2b for course credits. One participant was excluded due to very low Attention task accuracy (below 60%). The remaining participants’ data, N = 30 in each group, were included in the analyses (38 females, 21 males, 1 other gender, age mean = 18.98, SD = 1.21).

#### Apparatus and stimulus

Stimuli and equipment were identical to those of Experiment 2, except we replaced the sensory cues with word cues. The word cues were composed of color names (magenta, red, orange, yellow, green, blue) in uppercase English text (white, size = 32 pt) presented at screen centre.

#### Design and procedure

The design was identical to Experiment 2. Participants performed the tasks in either “high demand word cue” (HighWord) or “low demand word cue” (LowWord) condition. During the cue period, instead of color patches, participants were presented with the word cue. To ensure that participants could properly perform the high selection demand task with word cues where the cued and uncued colors for the Attention task could be fairly similar, we first trained them on the association of the word cues and their specific hues. This training involved a passive-viewed learning and a two-alternative forced choice testing where participants were given a color word and responded to which of the two similar color probes was the correct color. Participants repeated the learning and testing until they reached an accuracy of 90%. The probe color differences of this training task were determined based on the staircase thresholds from a pilot working memory task, the details of which are listed in Supplemental Table S2. Participants in the low selection demand group did not undergo any training, because training could artificially induce a strategy to maintain high precision of hues for word cues (the issue of training is further discussed in the following section).

Similar to Experiment 2, each participant completed 6 blocks, with each block containing 27 trials of the Search task and 27 trials of the Attention task. A total of 1.9% of the search trials were excluded from analyses due to RTs shorter than 200 ms or outside of 3 standard deviations from the mean RT for each condition within each participant.

#### Results and discussion

The overall performance of the Attention task and staircase thresholds confirm again successful manipulation on selection demand while controlling for task difficulty. The accuracy was 77% ~ 78% for both LowWord and HighWord conditions, and the staircased hue differences between cued and uncued colors in the high demand were on average less than 40°.

**Figure 4.**
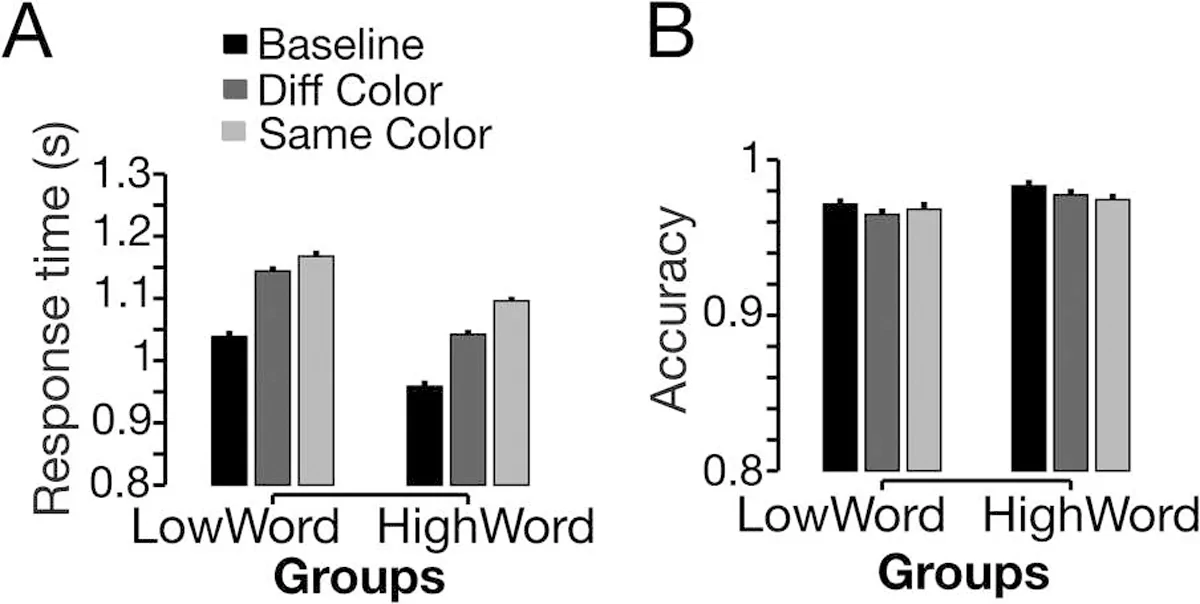
Search task performance of Experiment 3, including response time (A) and accuracy (B) for each distractor condition for LowWord and HighWord conditions. Within-subject error bars are plotted.

As shown in Figure 4, there was again a robust salience-driven capture effect shown in the Search RT. A 2 (baseline vs. different-color, within-subject) by 2 (LowWord vs. HighWord, between-subject) mixed-factor ANOVA revealed a significant main effect of distractor condition, *F*(1, 58) = 115.553, *p* <.001, $\eta_{p}^{2}=.666$, and no significant main effect of selection demand (*F*(1, 58) = 2.335, *p* = .132, $\eta_{p}^{2}=.039$) nor the interaction (*F*(1, 58) = 1.512, *p* = .224, $\eta_{p}^{2}=.025$). There was anecdotal evidence for no difference in salience-driven capture magnitude between LowWord and HighWord groups (independent samples t-test *t*(58) = 1.230, *p* = .224, *Cohen’s d* = 0.318, *BF*_*01*_ = 2.024).

For template-driven capture, we now observed different magnitudes of capture across conditions. From a 2 (same- vs. different-color, within-subject) by 2 (LowWord vs. HighWord, between-subject) mixed-factor ANOVA, we found a significant main effect of distractor color, *F*(1, 58) = 27.668, *p* <.001, $\eta_{p}^{2}=.323$, showing longer RT in the same-color compared to the different-color condition. Critically, there was also a significant interaction between selection demand and distractor color, *F*(1, 58) = 4.124, *p* = .047, $\eta_{p}^{2}=.066$, indicating a smaller template-driven capture in the LowWord than the HighWord condition. The main effect of selection demand was not significant, *F*(1, 58) = 2.122, *p* = .151, $\eta_{p}^{2}=.035$. Post hoc t-tests show that the HighWord group’s capture magnitude (RT difference between same-color and different-color) was significantly larger than zero (*t*(29) = 6.044, *p*_*holm*_ <.001, *Cohen’s d* = 1.103; *BF*_*10*_ > 100), whereas the LowWord group’s capture magnitude was only marginally significant (*t*(29) = 2.024, *p*_*holm*_ = .052, *Cohen’s d* = 0.370; *BF*_*10*_ = 1.156). The accuracy result did not show any significant main effects nor interaction, *Fs* ≤ 3.672, *ps* ≥.060, $\eta_{p}^{2}s\leq 0.060$.

We further compared the magnitude of template-driven capture in HighWord to that in the high demand sensory cue condition in Experiment 2 and found substantial evidence for no difference (independent samples *t*(58) = 0.575, *p* = .568, *Cohen’s d* = 0.259; *BF*_*01*_ = 3.317). This suggests that HighWord and high demand with sensory cues resulted in comparable template-driven capture.

We note that another difference between the HighWord and LowWord groups was the word-color association training used in the HighWord group only. This training procedure was necessary for participants to properly perform the task in the HighWord condition. For the LowWord group, however, such training was not necessary to perform the task and could instead induce response patterns unlikely to generalize to natural behaviour and thus it was not employed. Could this training procedure, by itself, produce the observed difference in template-driven capture? We ran an additional experiment in LowWord condition with the exact same training procedure as HighWord (see Supplemental Analysis and Figure S3 for details). This experiment yielded a similar magnitude of template-driven capture compared to that of the LowWord condition in Experiment 3. Taken together, all the data, we still observed a similar interaction between selection demand and distractor color, suggestive of a stronger sensory template due to heightened selection demand.

In summary, in contrast to the sensory cue format in Experiment 2, we found a reduced magnitude of template-driven capture in the LowWord condition compared to the HighWord condition, showing that selection demand modulates the strength of the sensory attentional template with non-sensory cue inputs. However, the template-driven capture effect was not completely eliminated in the LowWord condition, suggesting that there might still be some amount of sensory information in the template guiding attention.

## General discussion

In all three experiments, we found salience-driven capture effects, indicated by longer RTs in the different-color distractor condition than in the no-distractor baseline. As expected, this salience-driven capture effect was not influenced by selection demand or cue format, suggesting that it was a bottom-up and automatic process, and that the overall weighting of the color feature dimension was not influenced by selection demand or cue format (Muller et al., 2003).

In both Experiments 1 and 2, when sensory cues were used, we found reliable capture effects driven by attentional template content – indicated by longer RTs in the same-color condition than in the different-color distractor conditions – reflecting robust sensory information maintained in the attentional template with sensory cues. Selection demand did not influence the magnitude of this template-driven capture, indicating that the strength of the sensory templates was comparable when the color to be attended was given in its sensory format. Only in Experiment 3, when non-sensory cues were used, did selection demand modulate the strength of the sensory template, although the low-demand condition did not fully eliminate sensory content.

### Sensory cues promote reliable strength of sensory templates insensitive to selection demand

Our results suggest that when presented with sensory cue inputs, attentional templates are maintained with sensory information during the preparatory stage. Furthermore, the strength of sensory information is unaffected by levels of selection demand, even when there are explicit instructions to encourage non-sensory/verbal coding (Supplemental Figure S2A). This lack of modulation, replicated within our experiments, seems surprising and contradictory to some earlier studies on task demand. As introduced earlier, Olivers et al. (2006) manipulated memory demand by varying probe similarity in the recognition memory task, which is similar to how we manipulated attentional selection demand. In addition, they explicitly instructed participants to “remember the color verbally” when the memory probes were from different color categories, and to “remember the color visually” when the memory probes were all from the same color category. Their results showed that the working memory capture effect was completely eliminated in the “more verbal” condition, which is conceptually similar to the low selection demand condition in our Experiments 1 and 2, where we found reliable template-driven capture effects. We note that there are some critical differences between the studies. In our study, the Search and Attention tasks were randomly chosen on each trial, and overall task engagement was controlled by adaptive staircases, resulting in comparable behavioural performance between demand levels. In contrast, the working memory study used a sequential two-task design for each trial (search first, memory later), and the memory task difficulty was not controlled, leading to drastically different performance levels: the “more verbal” condition had an error rate of around 5%, whereas the “more visual” condition led to an error rate of around 40% (Olivers et al., 2006, Table 2). This large difference in memory performance, as well as predictable task sequence in each trial, could lead to an overall difference in task engagement between conditions, which in turn could drive the difference shown in working memory-driven capture. Further research is needed to isolate the effects of selection demand and task engagement to facilitate an equitable comparison between working memory and attentional template representations.

Although we did not find effects of selection demand on attentional capture, other studies have reported modulatory effects of selection demand, generally on the *precision* of attentional templates. For example, Geng et al. (2017) measured memory for the cued color using a secondary recall task and revealed a narrower tuning profile when target-distractor similarity was higher. Similarly, Kerzel (2019) measured attentional templates using the contingent capture paradigm while manipulating cue-target feature similarity and found a narrower profile of the capture effect for higher than for lower search demand. It is important to note, though, that in these designs, selection demand manipulation is often intertwined with overall task difficulty. Furthermore, the modulatory results do not indicate changes in the sensory format or the guiding power of attentional templates, but rather changes in the degree of precision (e.g., coarse vs. fine tuning profile, or lower vs. higher memory precision). The guiding strength of the feature template itself was not directly examined and compared between demand conditions in these studies.

It should be noted that we are not questioning the modulatory effect of selection demand on template precision. Had we measured precision in our design, we may well have found a sharpened tuning profile under high selection demand. Rather, we consider precision and guiding strength to reflect different attributes of attentional templates, such that a less precise template does not necessarily result in weakened guidance. A recent EEG finding suggests that such dissociation may exist at the neural level. Kerzel and Cong (2021) compared templates for singleton search and feature search and showed that while template precision measured behaviourally indeed varied between the two search modes, the N2pc – an ERP component commonly considered an indicator of attention guidance and location-based selection – remained unchanged between the two levels of search demand. Consistent with this study, our results provide behavioural evidence that the strength of sensory templates during attentional preparation, when generated from sensory cue inputs, is not susceptible to selection demand and is likely distinct from precision. These varying results across different measures of attentional templates suggest that templates have multiple attributes, which can be applied to multiple aspects or contexts of attentional processes in different ways.

### Non-sensory cue inputs activate sensory templates with modulated strength

Our results showed that when attentional cues were provided in word format, there were still sensory components in the templates which produced strong guidance to capture attention under high selection demand, similar to that produced by sensory cues. This suggests that the non-sensory cue of the target color elicits sensory information which is then maintained in the attentional template during preparation. This sensory information may be a result of active transformation from non-sensory inputs, or of coactivation where the template contains both sensory and non-sensory components. Moreover, the strength of this sensory component of template under high demand is comparable to the template directly generated from sensory cue inputs. In contrast, when the selection demand was low, we observed attenuated guiding power from the sensory information of the template.

Interestingly, even though we found a modulation effect of selection demand, we did not find a scenario where sensory information from attentional templates could be completely “removed.” The mechanism for activating sensory templates might be related to the observation that colors and their associated names used in natural languages are strongly connected (Regier et al., 2007). It is possible that after receiving the color-word cue, participants automatically retrieved the sensory information of that color from their long-term memory into an active state in working memory, which captured attention in the Search Task. For example, Sun et al. (2015) showed an automatic semantic-color association, such that colors associated with semantic concepts could automatically capture attention even though their task never required participants to make such association. That said, this automatic word-color association, if it existed, did not produce as strong attentional guidance under low demand as it did under high demand in our data, indicating that it alone has a weak effect. This can also explain the lack of word-produced sensory capture found in Baier and Ansorge (2019). In their study, participants were asked to maintain a constant search target, either a colored circle or a color name, throughout the experiment. They used the contingent capture paradigm to assess the representation of the search template. However, in contrast to Sun et al. (2015), they did not find color capture from word templates. This may be due to the characteristics of the contingent capture paradigm, which indirectly assesses top-down feature guidance by measuring its modulation of bottom-up spatial cueing effects. An automatic word-color association effect might not be strong enough to elicit such an interaction to be observed.

In our design, sensory information of the target color is ultimately necessary for the sustained attention task, but the fact that the target color already strongly guides attention during the preparatory stage reflects the active creation and maintenance of sensory templates under high selection demand. This finding, together with the strong sensory guidance found with sensory cues, aligns with the working memory literature. It is widely accepted that attentional templates are internal representations maintained in working memory (Desimone & Duncan, 1995; Duncan & Humphreys, 1989; Wolfe, 2021). One prominent theory of working memory, the sensory recruitment hypothesis (D’Esposito, 2007), proposes that WM maintenance recruits the same brain regions that are engaged in perception. Although there have been debates centring on the cortical loci of working memory maintenance (Scimeca et al., 2018; Xu, 2017), there is broad consensus that working memory of simple visual features can be maintained in sensory-like representations to support high-precision memory behaviours (see review in Adam et al., 2022). Our study provides behavioural evidence for the reliable existence of sensory templates during the preparatory stage (with varied strength in some circumstances), even when the attention-directing cue itself lacks sensory quality. It should be noted that we do not propose that attentional templates are represented *only* in a sensory format. For example, when maintaining color red as the sensory template, the word “red” and its semantics may also be activated and maintained. It is possible that the brain uses multiple forms of neural representations for working memory and attentional templates (Chen et al., 2025; Gong et al., 2022), and task contexts (e.g., searching for a redder-orange target among orange distractors) may encourage representations of relational information instead of precise perceptual representations (Becker et al., 2010; Geng et al., 2017).

An interesting question is why selection demand did not alter template strength with sensory cues but modulated strength with non-sensory cues. Here we provide some possible mechanisms. A critical difference in utilizing sensory versus non-sensory cues is activating the corresponding sensory information from non-sensory inputs. It seems plausible that selection demand modulates this activation process, whereby low selection demand may delay or obviate proactive activation during preparation, and/or activate a sensory template that is too low in precision to produce a reliable capture effect. All these possibilities would lead to a reduced amount or quality of sensory information in the LowWord condition, giving rise to a reduced guiding power of the sensory templates and a reduced capture in the search task. In contrast, for sensory cues, sensory information is already available with high fidelity regardless of demand level, and word-to-sensory transformation or activation is not necessary. The amount and quality of sensory content in the attentional templates are not downgraded, and the strength of guidance remains high to prepare for the upcoming feature selection task.

It should also be pointed out that color is the most common feature investigated in attention research, but the color feature may not generalize to other features (Nothdurft, 1993). With the working memory-driven capture paradigm, it has been shown that color has stronger guiding power than orientation (Teng & Kravitz, 2019) and shape (Soto et al., 2005), which may indicate differential characteristics of sensory templates across feature dimensions. A recent study isolated the color and form information from the picture-based cues and found that color and form features contributed to different aspects of the search process (Montalvo et al., 2024). In future experiments, it is important to investigate whether the current findings based on color templates and the influences of selection demand and cue format extend to other feature dimensions.

## Conclusions

In summary, our study shows that color templates for sustained feature-based attention are almost always maintained with some amount of sensory information and that selection demand modulates guiding strength of the templates only for non-sensory cues, possibly by influencing the activation from non-sensory inputs to sensory templates. Although template precision has been shown to be sensitive to selection demand in previous studies, in our study, with considerable methodological differences, the guiding strength of sensory information remained high with sensory cues. These findings raise further questions about how different attributes of attentional templates relate to one another and about how templates may function differently across task settings.

## Supplementary Material

Supp 1

Supplemental data for this article can be accessed online at https://doi.org/10.1080/13506285.2026.2687589.

## References

[R1] Adam KCS, Rademaker RL, & Serences JT (2022). Evidence for, and challenges to, sensory recruitment models of visual working memory. In Visual memory (pp. 5–25). Routledge. 10.4324/9781003158134-2

[R2] Addleman DA, Rajasingh R, & Störmer VS (2024). Attention to object categories: Selection history determines the breadth of attentional tuning during real-world object search. Journal of Experimental Psychology: General, 153(6), 1568–1581. 10.1037/xge0001575 38647455 PMC12007692

[R3] Alexander RG, & Zelinsky GJ (2011). Visual similarity effects in categorical search. Journal of Vision, 11(8), 9–9. 10.1167/11.8.9. PMC8409006 21757505

[R4] Anderson GM, Heinke D, & Humphreys GW (2010). Featural guidance in conjunction search: The contrast between orientation and color. Journal of Experimental Psychology: Human Perception and Performance, 36(5), 1108–1127. 10.1037/a0017179. 20718568

[R5] Baier D, & Ansorge U (2019). Investigating the role of verbal templates in contingent capture by color. Attention, Perception, & Psychophysics, 81(6), 1846–1879. 10.3758/s13414-019-01701-y. PMC6675783 30924054

[R6] Becker SI, Folk CL, & Remington RW (2010). The role of relational information in contingent capture. Journal of Experimental Psychology: Human Perception and Performance, 36(6), 1460–1476. 10.1037/a0020370. 20919781

[R7] Calder-Travis J, & Ma WJ (2020). Explaining the effects of distractor statistics in visual search. Journal of Vision, 20(13), 11. 10.1167/jov.20.13.11. PMC7746958 33331851

[R8] Chen Y, Liu T, Jia K, Theeuwes J, & Gong M (2025). Dual-format attentional template during preparation in human visual cortex. eLife, 13, RP103425. 10.7554/eLife.103425.2 41160433 PMC12571481

[R9] Cousineau D (2005). Confidence intervals in within-subject designs: A simpler solution to Loftus and Masson’s method. Tutorials in Quantitative Methods for Psychology, 1(1), 42–45. 10.20982/tqmp.01.1.p042.

[R10] de Groot F, Huettig F, & Olivers CNL (2016). When meaning matters: The temporal dynamics of semantic influences on visual attention. Journal of Experimental Psychology: Human Perception and Performance, 42(2), 180–196. 10.1037/xhp0000102. 26322686

[R11] Desimone R, & Duncan J (1995). Neural mechanisms of selective visual attention. Annual Review of Neuroscience, 18(1), 193–222. 10.1146/annurev.ne.18.030195.001205. 7605061

[R12] D’Esposito M (2007). From cognitive to neural models of working memory. Philosophical Transactions of the Royal Society B: Biological *Sciences*, 362(1481), 761–772. 10.1098/rstb.2007.2086. PMC2429995 17400538

[R13] Doyle A, Volkova K, Crotty N, Massa N, & Grubb MA (2025). Information-driven attentional capture. Attention, Perception, & Psychophysics, 87(3), 721–727. 10.3758/s13414-024-03008-z. PMC11965227 39971884

[R14] Dube B, & Golomb JD (2021). Perceptual distraction causes visual memory encoding intrusions. Psychonomic Bulletin & Review, 28(5), 1592–1600. 10.3758/s13423-021-01937-6. 34027621 PMC9255253

[R15] Duncan J, & Humphreys GW (1989). Visual search and stimulus similarity. Psychological Review, 96(3), 433–458. 10.1037/0033-295X.96.3.433. 2756067

[R16] Gardner JL, Merriam EP, Schluppeck D, & Larsson J (2018). MGL: Visual psychophysics stimuli and experimental design package (2.0). Zenodo. 10.5281/zenodo.1299497

[R17] Geng JJ, DiQuattro NE, & Helm J (2017). Distractor probability changes the shape of the attentional template. Journal of Experimental Psychology: Human Perception and Performance, 43(12), 1993–2007. 10.1037/xhp0000430. 28425732

[R18] Gong M, Chen Y, & Liu T (2022). Preparatory attention to visual features primarily relies on non-sensory representation. Scientific Reports, 12(1), 21726. 10.1038/s41598-022-26104-2. 36526653 PMC9758135

[R19] Huang L (2015). Color Is processed less efficiently than orientation in change detection but more efficiently in visual search. Psychological Science, 26(5), 646–652. 10.1177/0956797615569577. 25834029

[R20] Hulleman J (2020). Quantitative and qualitative differences in the top-down guiding attributes of visual search. Journal of Experimental Psychology: Human Perception and Performance, 46(9), 942–964. 10.1037/xhp0000764. 32378936

[R21] Team JASP. (2024). JASP (Version 0.95.0) [Computer software] https://jasp-stats.org/.

[R22] Kerzel D (2019). The precision of attentional selection is far worse than the precision of the underlying memory representation. Cognition, 186, 20–31. 10.1016/j.cognition.2019.02.001. 30739056

[R23] Kerzel D, & Cong SH (2021). Attentional templates Are sharpened through differential signal enhancement, not differential allocation of attention. Journal of Cognitive Neuroscience, 33(4), 594–610. 10.1162/jocn_a_01677. 33464161

[R24] Malcolm GL, & Henderson JM (2009). The effects of target template specificity on visual search in real-world scenes: Evidence from eye movements. Journal of Vision, 9(11), 8. 10.1167/9.11.8. 20053071

[R25] Maxfield JT, & Zelinsky GJ (2012). Searching through the hierarchy: How level of target categorization affects visual search. Visual Cognition, 20(10), 1153–1163. 10.1080/13506285.2012.735718. 23565048 PMC3616399

[R26] Montalvo DT, Rodriguez A, & Becker MW (2024). Isolating the impact of a visual search template’s color and form information on search guidance and verification times. Attention, Perception, & Psychophysics, 86(7), 2275–2288. 10.3758/s13414-024-02899-2. PMC11480184 38811488

[R27] Morey RD (2008). Confidence intervals from normalized data: A correction to Cousineau (2005). Tutorials in Quantitative Methods for Psychology, 4(2), 61–64. 10.20982/tqmp.04.2.p061.

[R28] Muller HJ, Reimann B, & Krummenacher J (2003). Visual search for singleton feature targets across dimensions: Stimulus- and expectancy-driven effects in dimensional weighting. Journal of Experimental Psychology: Human Perception and Performance, 29(5), 1021–1035. 10.1037/0096-1523.29.5.1021. 14585020

[R29] Nothdurft H-C (1993). The role of features in preattentive vision: Comparison of orientation, motion and color cues. Vision Research, 33(14), 1937–1958. 10.1016/0042-6989(93)90020-W. 8249312

[R30] Olivers CNL, Meijer F, & Theeuwes J (2006). Feature-based memory-driven attentional capture: Visual working memory content affects visual attention. Journal of Experimental Psychology: Human Perception and Performance, 32(5), 1243–1265. 10.1037/0096-1523.32.5.1243. 17002535

[R31] Prins N, & Kingdom FA (2018). Applying the model-comparison approach to test specific research hypotheses in psychophysical research using the palamedes toolbox. Frontiers in Psychology, 9, 1250. 10.3389/fpsyg.2018.01250 30083122 PMC6064978

[R32] Reeder RR, & Peelen MV (2013). The contents of the search template for category-level search in natural scenes. Journal of Vision, 13(3), 13–13. 10.1167/13.3.13. 23750015

[R33] Regier T, Kay P, & Khetarpal N (2007). Color naming reflects optimal partitions of color space. Proceedings of the National Academy of Sciences, 104(4), 1436–1441. 10.1073/pnas.0610341104. PMC1783097 17229840

[R34] Scimeca JM, Kiyonaga A, & D’Esposito M (2018). Reaffirming the sensory recruitment account of working memory. Trends in Cognitive Sciences, 22(3), 190–192. 10.1016/j.tics.2017.12.007. 29475635

[R35] Soto D, Heinke D, Humphreys GW, & Blanco MJ (2005). Early, involuntary Top-down guidance of attention from working memory. Journal of Experimental Psychology: Human Perception and Performance, 31(2), 248–261. 10.1037/0096-1523.31.2.248. 15826228

[R36] Sun SZ, Shen J, Shaw M, Cant JS, & Ferber S (2015). Automatic capture of attention by conceptually generated working memory templates. Attention, Perception, & Psychophysics, 77(6), 1841–1847. 10.3758/s13414-015-0918-1. 25944451

[R37] Teng C, & Kravitz DJ (2019). Visual working memory directly alters perception. Nature Human Behaviour, 3(8), 827–836. 10.1038/s41562-019-0640-4. 31285620

[R38] The Mathworks Inc. (2015). MATLAB (Version 8.6.0 (R2015b)) [Computer software] https://www.mathworks.com.

[R39] Treisman AM, & Gelade G (1980). A feature-integration theory of attention. Cognitive Psychology, 12(1), 97–136. 10.1016/0010-0285(80)90005-5. 7351125

[R40] Vickery TJ, King L-W, & Jiang Y (2005). Setting up the target template in visual search. Journal of Vision, 5(1), 8. 10.1167/5.1.8. 15831069

[R41] Wolfe JM (2012). When do I quit? The search termination problem in visual search. In Dodd MD, & Flowers JH (Eds.), The influence of attention, learning, and motivation on visual search (pp. 183–208). Springer. 10.1007/978-1-4614-4794-8_8. PMC3979292 23437634

[R42] Wolfe JM (2021). Guided search 6.0: An updated model of visual search. Psychonomic Bulletin & Review, 28(4), 1060–1092, 10.3758/s13423-020-01859-9. 33547630 PMC8965574

[R43] Xu Y (2017). Reevaluating the sensory account of visual working memory storage. Trends in Cognitive Sciences, 21(10), 794–815. 10.1016/j.tics.2017.06.013. 28774684

[R44] Yu X, Johal SK, & Geng JJ (2022). Visual search guidance uses coarser template information than target-match decisions. Attention, Perception, & Psychophysics, 84(5), 1432–1445. 10.3758/s13414-022-02478-3. PMC9232460 35474414

