# Supplementary material for: The guiding strength of sensory templates during preparatory stage of sustained feature-based attention
Source: Vis cogn. Author manuscript; Available in PMC 2026 Sep 12. (PMC13565366; doi:10.1080/13506285.2026.2687589)
Supplement: Supplementary file 1 [file NIHMS2197336-supplement-Supp_1.pdf]

The guiding strength of sensory templates during preparatory stage of sustained feature-based attention

Supplemental Materials

Xiaoli Zhang and Taosheng Liu

Department of Psychology, Michigan State University

Corresponding author:

Xiaoli Zhang (zhan2208@msu.edu)

Table S1 RGB values of target colors used in each experiment.

Target Color	RGB values
Experiment 1	
Red	(255, 28, 100), (255, 42, 81), (255, 57, 62)
Yellow	(255, 121, 8), (255, 145, 8), (216, 164, 8)
Green	(142, 186, 8), (73, 196, 34), (18, 202, 90)
Blue	(18, 206, 180), (18, 196, 255), (49, 156, 255)
Experiment 2 & 3	
Magenta	(255, 12, 132)
Red	(255, 50, 69)
Orange	(255, 100, 8)
Yellow	(245, 152, 8)
Green	(73, 196, 34)
Blue	(18, 173, 255)

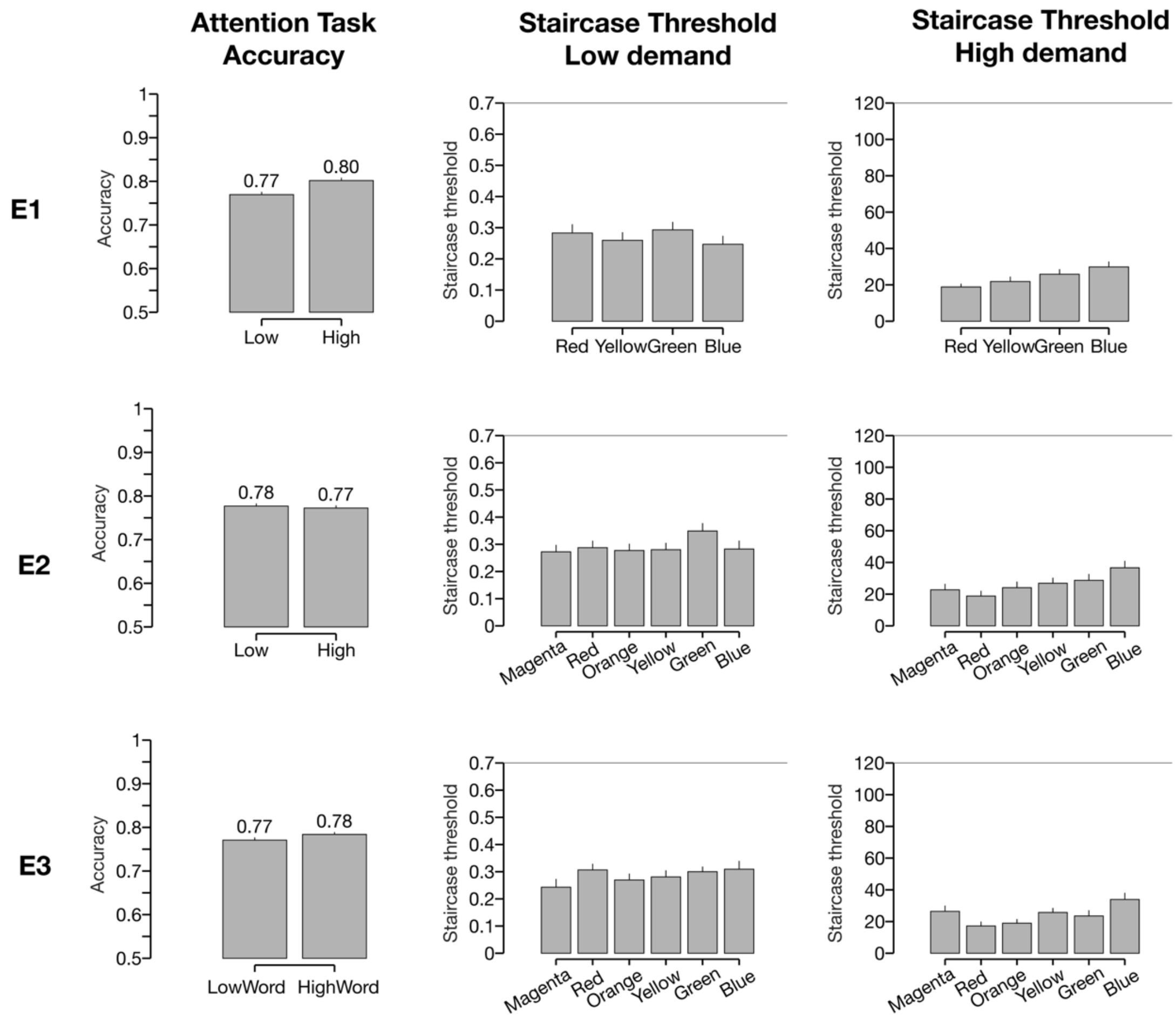

Figure S1 Attention task performance for Experiment 1 (1st row), Experiment 2 (2nd row), and Experiment 3 (3rd row). Left column: accuracy of the Attention task in low and high demand conditions respectively. Middle column: luminance thresholds for low demand condition separately for each color category (for reference, luminance change magnitude used in high demand condition is shown by the horizontal line at 0.7). Right column: angular thresholds for high demand condition separately for each color category (for reference, the angular distance between the cued and uncued color in low demand condition is shown by the horizonal line at 120°). Within-subject error bars are plotted.

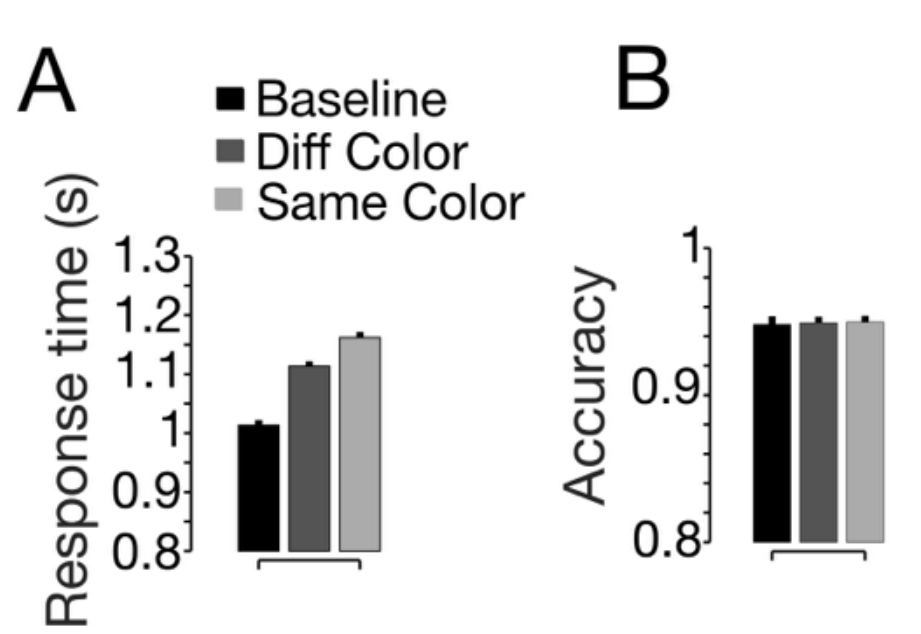

Figure S2 (A) Search RT results of Experiment 2 follow-up with verbal coding instruction, low demand visual cue. There was a significant template-driven capture effect as reflected by the paired sample t-test between same-color and different-color

distractor conditions, t(29) = 3.801, p < .001, Cohen's d = 0.694. More critically, the magnitude of this capture (i.e., same color minus different color condition) was not different from those in Experiment 1 (independent samples t-test compared to high demand, , t(58)=0.079, p=.938, Cohen's d=0.020, BF_{01}=3.801; compared to low demand, , t(58) = 0.825, p = .413, Cohen's d = 0.213, BF_{01} = 2.863), and from those in Experiment 2 (independent samples t-test compared to high demand, t(58)=0.820, p=.416, Cohen's d=0.212, BF_{01}=2.874; compared to low demand, t(58)=0.045, p=.965, Cohen's d=0.011, BF_{01}=3.808).
(B) Search accuracy results. There was no difference between baseline and different-color conditions, t(29) = 0.174, p = .863, Cohen's d = 0.032, BF_{01} = 5.072; no difference between different-color and same-color conditions, t(29) = 0.093, p = .927, Cohen's d = 0.017, BF_{01} = 5.123.
Within-subject error bars are plotted.

Table S2 The memory probe differences on the color wheel used for color-word training in Experiment 3 (HighWord condition).

Target Color	Angular difference of the memory probes on the color wheel (°)
Magenta	15
Red	15
Orange	20
Yellow	20
Green	20
Blue	30

Supplemental Experiment: LowWord with word-color training

In an effort to isolate the effect of training, we ran an additional experiment in LowWord condition with the exact same training procedure as HighWord (Figure S3). Comparing this LowWord-train (pre-registered N=30) group with the original LowWord group, we found no significant main effect of training nor interaction between training and distractor feature (same- vs. different-color) on RTs, *Fs* £ 2.159, *ps* ^{3} .147, h_p^2s £ 0.036. Specifically, there was substantial evidence showing that the magnitudes of template-driven capture were not different between LowWord-train (28ms on average) and LowWord (24ms on average), $t(58) = 0.255$, $p = .800$, *Cohen's d* = 0.066; BF_{01} = 3.708. Hence, training did not by itself produce a reliable modulation of capture in the LowWord condition. When comparing LowWord-train with HighWord, we found a marginally significant interaction between group and distractor feature on RTs, $F(1, 58) = 3.382$, $p = .071$, $h_p^2 = .055$, which is qualitatively similar to our original comparison. Given the lack of difference between the two LowWord conditions and the potential lower statistical power due to between-subject comparisons, we combined these two low demand groups to obtain a more robust estimate of the capture effect. When we compared this combined LowWord group with the HighWord group, and the result showed a significant interaction between demand and distractor feature on RTs, $F(1, 88) = 4.682$, $p = .033$, $h_p^2 = .051$.

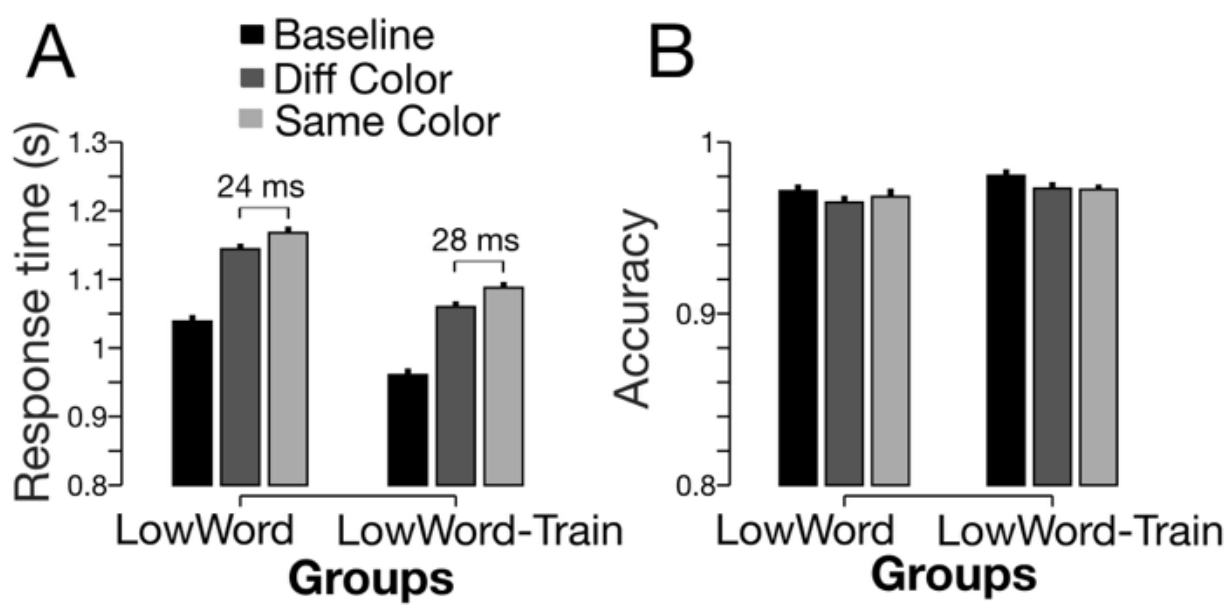

Figure S3 (A) Search RT result comparing LowWord (reproduced from Figure 4A) and LowWord-train groups. (B) Search accuracy results comparing LowWord (reproduced from Figure 4B) and LowWord-train groups.

Taken together, although the evidence from the LowWord-train data alone was not very strong, the combined results from HighWord, LowWord, and LowWord-train give rise to a coherent pattern that the training procedure is not a major factor to influence the magnitude of template-driven capture, and selection demand still exerts a modulatory effect on capture after considering the training factor. However, we acknowledge that this reasoning is based on statistical evidence alone and improved methods (e.g., fully-crossed experimental designs) can further strengthen the conclusion.
